# Effects of Yellow Mealworm (*Tenebrio molitor*) on Growth Performance, Hepatic Health and Digestibility in Juvenile Largemouth Bass (*Micropterus salmoides*)

**DOI:** 10.3390/ani13081389

**Published:** 2023-04-18

**Authors:** Haijie Chen, Jiao Yu, Xudong Ran, Jiaxuan Wu, Yongjun Chen, Beiping Tan, Shimei Lin

**Affiliations:** 1 College of Fisheries, Southwest University, Chongqing 400715, China; 2 Key Laboratory of Freshwater Fish Reproduction and Development (Ministry of Education), Southwest University, Chongqing 400715, China; 3 College of Fisheries, Guangdong Ocean University, Zhanjiang 524088, China

**Keywords:** *Tenebrio molitor*, growth, liver health, digestibility, *Micropterus salmoides*

## Abstract

**Simple Summary:**

Nowadays, owing to its limited availability and high cost, fish meal is no longer an affordable protein source in fish feed. Therefore, it is necessary to find new sustainable protein sources to replace fishmeal in the diet of fish or in aquafeeds. Recently, yellow mealworm (*Tenebrio molitor*) has been widely used as a protein source in the formulated feed of broilers, pigs, shrimp and fish, due to its high protein content, excellent amino acid profile and abundant functional substances. In this study, we found that yellow mealworm meal has high digestibility in largemouth bass (*Micropterus salmoides*) and an appropriate level (less than 19.52%) in the diet can promote growth and improve liver health in largemouth bass. Therefore, we assume that utilization of yellow mealworm in the diet of largemouth bass as a protein source is feasible.

**Abstract:**

This study investigated the effects of yellow mealworm meal (TM) on growth performance, hepatic health and digestibility in juvenile largemouth bass (*Micropterus salmoides*). The fish were fed with the basic feed and the test feed (70% basic feed and 30% raw materials) containing Cr_2_O_3_, and feces were collected for digestibility determination. The fish were fed with five isonitrogenous (47% crude protein) and isolipidic (13% crude lipid) diets, in which fishmeal (FM) was replaced with 0% (TM0), 12% (TM12), 24% (TM24), 36% (TM36) and 48% (TM48) TM. The fish were reared in cylindrical plastic tanks in a recirculating aquaculture system for 11 weeks. The apparent digestibility coefficients (ADC), of dry matter, crude protein and crude lipid, in largemouth bass of TM were 74.66%, 91.03% and 90.91%, respectively. The ADC of total amino acid (TAA) of TM in largemouth bass was 92.89%, and the ADC of essential amino acid (EAA) in TM in largemouth bass was 93.86%. The final body weight (FBW), weight gain rate (WGR) and specific growth rate (SGR) in the TM24 group were significantly higher than those in other groups. Similarly, the highest mRNA expression levels of hepatic protein metabolism genes (*pi3k*, *mtor*, *4ebp2* and *got*) and antioxidant enzyme (glutathione peroxidase, Gpx; catalase, Cat) activities were observed in the TM24 group. Moreover, the expression levels of anti-inflammatory factors (*il-10* and *tgf*) in liver were up-regulated and the expression levels of pro-inflammatory factors (*il-8* and *il-1β*) in liver were down-regulated. Quadratic regression model analysis, based on weight gain rate (WGR) against dietary TM level, indicated that the optimum level of dietary TM replacing FM in largemouth bass diet was 19.52%. Appropriate replacement levels (less than 36%) of FM by TM in the diets can enhance the antioxidant capacity and immunity of largemouth bass. However, high levels of FM substitution with TM (more than 48%) in the feeds can damage the liver health and inhibit the growth of largemouth bass. Notably, largemouth bass has high ADC and high utilization of TM, which indicates that it is feasible to use TM as feed protein source for largemouth bass.

## 1. Introduction

Fishmeal (FM) has been a crucial protein source for the aquaculture feed industry due to its high protein content, excellent amino acid profile and high digestibility [1]. However, with the rapid development of aquaculture, FM is an unaffordable protein source for fish feed owing to its high price and limited supply. Therefore, there is a need to find a sustainable and low-cost protein source for the feed industry. In recent years, insects have been recognized as a sustainable source of nutrients in the formulated feed of livestock and fish due to their high nutritional value, high feed conversion efficiency, rapid growth, short breeding cycle and ease of artificial large-scale breeding [2,3,4].

Among insects, yellow mealworm meal (TM) is allowed to be used as a fish feed ingredient by the European Union [5] and is considered a high quality protein source due to its high nutritional value (44–69% crude protein, 23–47% crude lipid), excellent amino acid profile, high digestibility and abundant functional substances such as chitin, antimicrobial peptide and antifreeze protein [6,7,8]. At present, TM is widely used as a protein source in the formulated feed of broilers [9], pigs [10], shrimp [11] and fish [12]. In addition, the supplementation of dietary TM at an appropriate level can enhance the growth and improve the health of fish [13,14,15]; furthermore, for some fish, such as rockfish (*Sebastes schlegeli)*, TM has a high digestibility [16]. The optimal level of TM in the feed is related to the TM processing method, animal species and growth stage. Largemouth bass (*Micropterus salmoides*) is one of the most important aquaculture fish in China and is popular among the public for its fresh meat and high nutritional value. The aquaculture production of largemouth bass in China reached 702,000 tons in 2021 [17]. However, with the rapid development of intensive aquaculture in recent years, liver disease in largemouth bass has become increasingly prominent [18], which has restricted the development of the industry. Therefore, this study aimed to evaluate the effects of replacing FM with TM on growth performance and hepatic health in largemouth bass, and to determine the digestibility in largemouth bass of TM.

## 2. Materials and Methods

### 2.1. Experimental Diets and Design

The coefficients of total tract apparent digestibility of the diets were measured using the indirect method proposed by Cho et al. [19]. The test feed was prepared with 70% basic feed and 30% raw materials, and 0.5% chromium trioxide (Cr_2_O_3_) was used as an inert indicator to determine the apparent digestibility of TM. Seventy-two largemouth bass (158.9 ± 1.7 g) were divided into six 200 L cylindrical plastic tanks (three replicate tanks per diet, twelve fish per tank). After 10 days of feeding with the basic feed (TM0) in the indoor circulation culture system, the basic feed and the test feed containing Cr_2_O_3_ were fed by hand twice (08:30 and 18:00) daily. The feces were collected by siphoning, six hours after feeding, and stored at −20 °C until analysis.

The amino acid (AA) composition and nutrient levels of both TM and FM are shown in Table 1. Five isonitrogenous (47% crude protein) and isolipidic (13% crude lipid) diets were formulated by replacing 0 (control, TM0), 12% (TM12), 24% (TM24), 36% (TM36) and 48% (TM48) FM with TM (Table 2). The experimental diets were supplemented with a combination of lysine and methionine to match the EAA profiles of the control diet. All ingredients were sieved using a 177 µm mesh-sized sieve before mixing. After the pelleted feed, with particle size of 3 mm, was made using a dry power press MUZL180 (Muyang Group, Yangzhou, China), it was naturally air-dried and stored at 4 °C for later use.

### 2.2. Experimental Procedures

Largemouth bass juveniles used in this experiment were obtained from Chongqing Three Gorges Ecological Fishery Co., LTD, Chongqing, China. Prior to the feeding trial, fish were placed in a cylinder with a volume of 400 L to acclimatise to a new environment and fed a commercial feed (Guangzhou Haida Feed Co., LTD., Guangzhou, China) for ten days. Then the fish were fasted for 24 h; largemouth bass (initial weight: 11.00 ± 0.25 g) were randomly distributed into 15 cylindrical plastic tanks (capacity: 200 L), at a density of 25 fish per tank, for the feed trial. Each group was randomly assigned to three tanks. The fish were reared in 200 L cylindrical plastic tanks in a indoor recirculating aquaculture system. The water source for the aquaculture was fully aerated tap water. Fish were fed twice daily (08:30 and 18:00), until apparent satiation, for 11 weeks. During aquaculture, the photoperiod was 12 L:12 D and the illumination time was from 08:00 to 20:00. In addition, the water temperature ranged from 25 to 29 °C, at pH 6.7–7.2, dissolved oxygen ≥ 7.0 mg/L, ammonia nitrogen < 0.1 mg/L, and nitrite < 0.01 mg/L.

### 2.3. Sample Collection

At the end of feed trial, the fish were fasted for 24 h and were then weighed and counted separately after being anesthetized with 0.01% MS-222 (Sigma, Wappingers Falls, New York, USA) for the growth indices (FBW, WGR, SGR) assay. After the body length and weight of 18 fishes (6 fish per tank) were measured for condition factor (CF), the viscera and livers were dissected on an ice tray and weighed for viscerosomatic index (VSI) and hepatosomatic index (HSI). Moreover, the central part of the liver was collected for histology, liver antioxidant ability and quantitative real time PCR.

### 2.4. Chemical Analysis

Total lipid in liver was measured following the method of Bligh and Dyer [20] and all chemical composition analysis of diets and whole body were conducted by Association of Official Analytical Chemists standard methods [21]. Moisture was determined by oven drying to a constant weight at 105 °C; crude protein was determined by measuring nitrogen (N × 6.25) by the Kjeldahl method; lipid was measured by ether extraction using Soxtec; ash was determined by combustion at 550 °C for 12 h in a muffle furnace. AA content was determined using an automatic AA analyzer (L-8900, Hitachi, Tokyo, Japan) after using acid hydrolysis to pre-treat samples (tryptophan was not detected).

Liver sections, treated as described by Su et al. [22], were stained with hematoxylin and eosin (H&E) before being observed under a light microscope (OLYMPUS, DP73, Nikon Corporation, Tokyo, Japan).

### 2.5. Liver Antioxidant Ability

The liver tissue was weighed and cut into small pieces; normal saline (9 times the weight of the liver tissue) and enzyme-free beads were added to an enzyme-free tube, which was then ground in a high-speed tissue grinder (KZ-11, Wuhan Servicebio Technology Co., Ltd., Wuhan, China) for 10 min. After centrifugation at 4000× *g* for 10 min, the supernatant was extracted and determined. The activities of total superoxide dismutase (T-Sod), catalase (Cat), glutathione peroxidase (Gpx) and the content of malondialdehyde (MDA) in liver were determined using commercial kits (Nanjing Jiancheng Bioengineering Institute, Nanjing, China).

### 2.6. Quantitative Real-Time PCR Analysis

Total RNA from liver was extracted using RNAiso Plus reagent (Takara, Kyoto, Japan) and the concentration and purity of total RNA were determined with a NanoDrop 2000 (Thermo, Waltham, MA, USA). Then, complementary DNA (cDNA) was synthesized from total RNA using the reverse transcription kit (Takara, Japan) and stored at −20 °C for quantitative real-time PCR, which was performed using the CFX96TM real-time system (Bio-Rad, Hercules, Carlifornia, USA). The primer sequences used for quantitative real-time PCR are shown in Table 3 and the relative quantification of gene expression was analyzed using the 2^−ΔΔCt^ method [23].

### 2.7. Calculations and Statistical Methods

The following variables were calculated with the following formulas:

ADC of dry matter in feed (%) = 100 × (1 − Cr_2_O_3_ in feed/Cr_2_O_3_ in feces) (1)

ADC of nutrients in feed (%) = 100 × [1 − (Cr_2_O_3_ in feed × nutrients in feces)/(Cr_2_O_3_ in feces/nutrients in feed) (2)

ADC of nutrients in TM (%) = ADC of nutrients in the test feed + [0.7 × nutrients in the base feed/(0.3 × nutrients in TM)] × (ADC of nutrients in the test feed − ADC of nutrients in the base feed)(3)

All data were analyzed using SPSS version 17.0 and presented as the mean ± standard error (SE). One-way ANOVA was used to analyze the differences between the groups before Tukey’s multiple comparison, and the level of significance was set at *p* < 0.05. Furthermore, orthogonal polynomial comparisons were used to determine whether the effects were linear or quadratic.

## 3. Results

### 3.1. Apparent Digestibility Coefficient of Tenebrio molitor

The ADCs of dry matter, crude protein and crude lipid in TM were 74.66%, 91.03% and 90.91%, respectively, in largemouth bass (Table 4). The ADCs of individual AA in TM were different, but all above 90%. Moreover, the ADCs of Lys and Met were above 95% (Table 5).

### 3.2. Growth Performance

**Figure 1:**
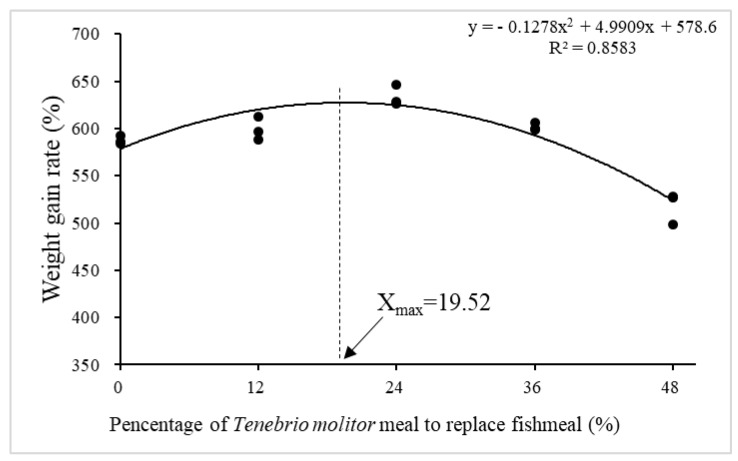
The relationship between WGR and dietary TM replacement level in *Micropterus salmoides.*

As shown in Table 6, the survival rate of all groups was 100% during the culture period. Significant linear and quadratic effects of FM replacement by TM were observed in FBW, WGR, SGR and LRR (*p* < 0.05). The PER and PRR showed a quadratic relationship with dietary TM levels (*p* < 0.05). The highest FBW, WGR and SGR were observed in the TM24 group (*p* < 0.05). The lowest LRR was observed in the TM36 group (*p* < 0.05). No significant effects of dietary TM on PER, FR and RCR were found (*p* > 0.05). Quadratic regression model analysis based on WGR against dietary TM replacement level indicated that the optimum replacement level in the diets was 19.52% (Figure 1).

### 3.3. Body Composition

No significant effects of dietary TM levels on CF, VSI, moisture and ash were observed (Table 7, *p* > 0.05). The HSI and crude fat showed a significant negative linear trend with the increasing TM levels (*p* < 0.05), and the HSI and crude fat of the TM48 group were significantly lower than those of the TM0 group (*p* < 0.05). The crude protein showed a linear and quadratic relationship with increasing dietary TM levels (*p* < 0.05). The crude protein of the TM0 group was significant lower than that of other groups (*p* < 0.05).

### 3.4. Liver Protein Metabolism

**Figure 2:**
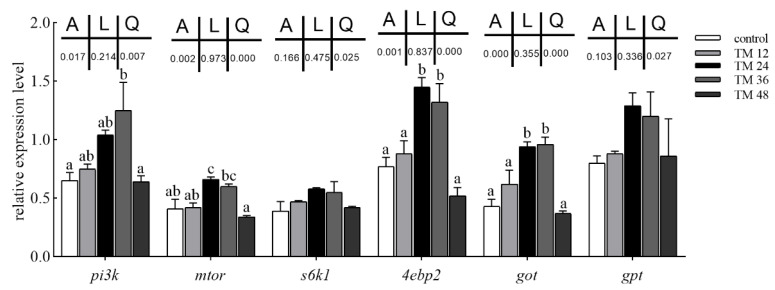
Effects of different levels of TM, instead of FM, on the expression of protein metabolism genes in the liver of *Micropterus salmoides.* Values with different letters represent significant differences in Tukey’s test (*p* < 0.05). A = the variance analyzed by one-way ANOVA; L = linear trend analyzed by orthogonal polynomial contrasts; Q = quadratic trend analyzed by orthogonal polynomial contrasts.

As shown in Figure 2, there were significant quadratic relationships between the expression levels of protein metabolism genes (*pi3k*, *mtor*, s6k1, *4ebp2*, *got* and *gpt*) and dietary TM level (*p* < 0.05). The mRNA expression levels of *pi3k*, *mtor*, *4ebp2* and *got* in the TM24 group were significantly higher than those in the TM0 group (*p* < 0.05).

### 3.5. Liver Antioxidant Capacity and Immunity

**Figure 3:**
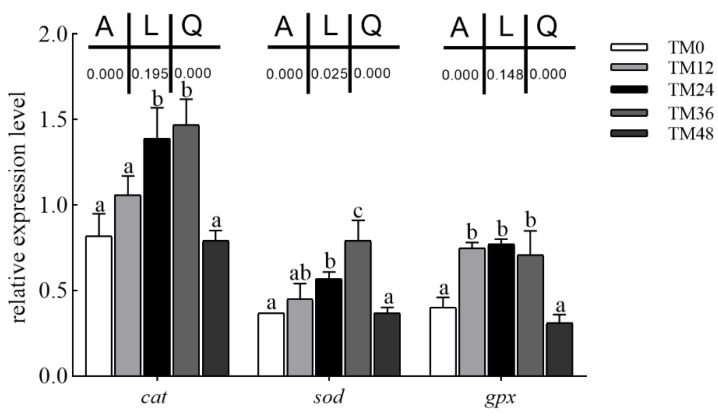
Effects of different levels of TM, instead of FM, on the expression of antioxidant genes in the liver of *Micropterus salmoides.* Values with different letters represent significant differences in Tukey’s test (*p* < 0.05). A = the variance analyzed by one-way ANOVA; L = linear trend analyzed by orthogonal polynomial contrasts; Q = quadratic trend analyzed by orthogonal polynomial contrasts.

There were significant quadratic relationships between the activities of Cat, Gpx and content of MDA in liver and dietary TM levels (*p* < 0.05; Table 8). The activities of Cat and Gpx in the TM24 group livers were significantly higher than those in the TM0 group (*p* < 0.05). Accordingly, A significant quadratic relationship was found between the expression of antioxidant genes (*cat*, *sod and gpx)* in the liver and dietary TM levels (*p* < 0.05, Figure 3).

**Figure 4:**
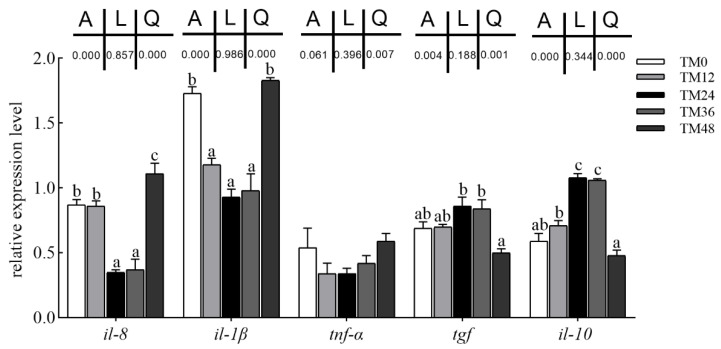
Effects of different levels of TM, instead of FM, on the expression of immune-related genes in the liver of *Micropterus salmoides.* Values with different letters represent significant differences in Tukey’s test (*p* < 0.05). A = the variance analyzed by one-way ANOVA; L = linear trend analyzed by orthogonal polynomial contrasts; Q = quadratic trend analyzed by orthogonal polynomial contrasts.

In Figure 4, there were significant quadratic relationships between the expression levels of immune-related genes (*il-8*, *il-1β*, *tnf-α*, *tgf* and *il-10*) in the liver and dietary TM levels (*p* < 0.05). The expression levels of liver proinflammatory cytokines (*il-8* and *il-1β*) in the TM24 group were significantly lower than those in the TM0 group (*p* < 0.05), whereas the expression levels of anti-inflammatory cytokines (*il-10* and *tgf*) in the TM24 group were significantly higher than those in the TM0 group (*p* < 0.05).

### 3.6. Liver Histology

**Figure 5:**
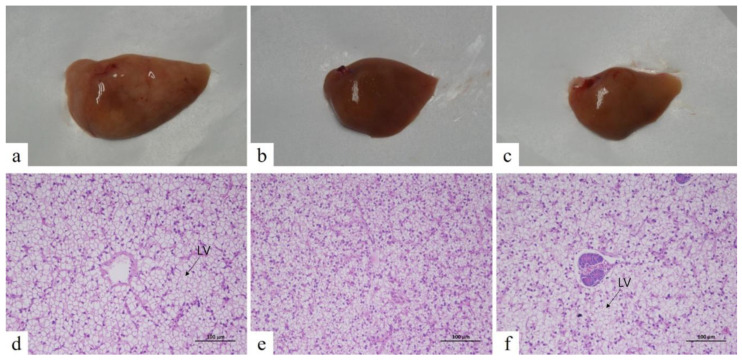
Effects of different levels of TM, instead of FM, on liver color and tissue structure in *Micropterus salmoides.* (**a**) Macrograph of liver in TM0 group; (**b**) macrograph of liver in TM24 group; (**c**) macrograph of liver in TM48 group; (**d**) paraffin section of liver in control group (200×); (**e**) paraffin section of liver in TM24 group (200×); (**f**) paraffin section of liver in TM48 group (200×). LV, lipid vacuole.

The livers of TM0 group were white and swollen, and many hepatocytes were vacuolated (Figure 5). The livers and their nuclei in the TM24 group were reddish and neatly arranged, respectively. Moreover, many vacuolations were observed in TM48 group hepatocytes.

## 4. Discussion

Fish can only utilize the digestible nutrients in their feed, therefore, the digestible raw ingredients in the diets can improve the utilization efficiency of the compound feed. The ADC of nutrients can reflect the utilization rate of nutrients in feed by organisms. In the present study, the ADCs in largemouth bass of dry matter, crude protein and crude fat of TM were 74.66%, 91.03% and 90.91%, respectively. Similar results were observed in European sea bass (*Dicentrarchus labrax*), which indicated the ADCs of dry matter, crude protein and crude fat in TM were 85.2%, 89.2%, and 94.5% [6]. Gasco et al. [24] found that the ADC of the crude protein in seabass diets containing 25% TM was significantly higher than in the FM group. However, the ADC of crude protein in the diets of rainbow trout fed diets supplemented with 50% TM was significantly lower than in FM group [25]. In addition to dry matter, the ADC of TM in largemouth bass was similar to that of blood meal and poultry by-product meal, and higher than that of meat bone meal [26]. These results indicated that the ADC of TM raw materials is affected by the species, size, growth stage of the fish and the TM processing technology . Protein digestibility is closely related to AA digestibility; thus, it is more meaningful to determine the AA digestibility of protein sources. In the current study, the ADC of AA in TM was more than 90%; in particular, the ADCs of lysine and methionine were higher than 95%. A previous study found that the EAA digestibility of FM in largemouth bass was higher than 90% [26], which is similar to the EAA digestibility of TM to largemouth bass to in this study. In the case of the ADC, TM could be well digested and absorbed by largemouth bass.

The present study demonstrated that dietary FM could be replaced with TM up to 19.52% according to the quadratic regression model analysis of WGR against dietary TM replacement level. Accordingly, the optimal supplemental level of TM in largemouth bass diet was 7.80%. However, replacement of FM at levels above 36% negatively affected largemouth bass growth. Studies have found that the replacement of 20% FM by TM does not affect the growth performance of flounder (*Paralichthys olivaceus*), but when the replacement level of FM exceeds 40%, it can inhibit fish growth [27]. Similarly, replacing 25% FM with TM does not affect the growth performance of European perch (*Perca fluviatilis)*; however, replacing more than 50% of FM does inhibit its growth [28]. Substitution of 30% FM with TM does not affect the growth performance of large yellow croaker (*Larimichthys crocea*), but substitution of FM at levels over 45% reduces feed efficiency ratio and PER, which inhibits the growth of large yellow croaker [29]. These results are similar to the results of this study, which indicates that it is feasible to replace FM with an appropriate amount of TM. However, replacement of FM with a high proportion (about 67%) of TM does not affect the growth of rainbow trout *(Oncorhynchus mykiss)* [25]. In addition, TM can completely replace FM (65% FM content) to promote the growth of red sea bream (*Pargus major*) [12]. The differences in these results may be related to the differences in the levels of nutrients, freshness of FM, amount of TM, fish species, feed formula and other factors.

The present study found that the PER and PRR had a quadratic relationship with dietary TM levels, and PER in the TM24 group was significantly higher than in the TM0 and TM48 groups; however, both FR and FCR in the TM24 group were lower than in the TM0 group or TM48 group, indicating that an appropriate level of TM in the diet can promote the growth of largemouth bass by improving feed utilization and protein deposition. This may be related to bioactive compounds in TM, such as chitin, antimicrobial peptide and β-glucanase [30,31]. Chitin has been shown to enhance immune response and resistance [32]. AMP is considered a possible anti-infective agent to reduce infectivity [33]. Similarly, β-glucanase has been shown to increase resistance against infection [34]. These bioactive compounds can improve health and growth by enhancing body immunity and anti-infection mechanisms. On the contrary, the replacement of 48% FM by TM decreased feed utilization efficiency and inhibited its growth, which may be related to the high content of chitin. Excessive chitin interferes with the digestion and absorption of the feed by binding AA to form indigestible complexes [35]. At present, chitinase activity has been found in fish; chitinase can degrade insects and zooplankton containing chitin in the gastrointestinal tract of fish to help digestion [36]. Piccolo et al. [37] found that chitin in the diet may be partially degraded by the endogenous chitinase of gilthead sea bream *(Sparus aurata).* However, as the TM levels increase, the amount of chitin in the digestive system of the fish increases, suggesting that the content of chitinase may be not sufficient to digest the increased chitin content. No studies have reported the discovery of chitinase in the digestive tract of largemouth bass, and the mechanism of chitin digestion in largemouth bass needs to be further explored.

Liver is the main site of nutrient metabolism in fish and it regulates the metabolism of three major nutrients. Moreover, it is particularly important in the maintenance of normal physiological function. This study found that dietary TM had a certain effect on the liver structure of largemouth bass. Compared with the TM0 group, the liver and their nuclei in the TM24 group were redder and more neatly arranged, respectively, which may be related to antibacterial and antifungal substances in the insect cuticle that could prevent liver disease, stress and tumor [38]. In this study, the activities of antioxidant enzymes (Cat and Gpx) and the expression levels of antioxidant genes (*cat*, *sod* and *gpx*) in liver increased significantly with increasing TM replacement levels, and then decreased, whereas the content of MDA decreased first and then increased. This indicates that an appropriate level of TM in the diet can improve the antioxidant capacity of largemouth bass, which may be due to the fact that chitin, **c**hitosan (a product of chitin after certain chemical process), and their derivatives have free radical scavenging activities [39]. However, the high level of substitution will lead to oxidative damage. This is consistent with previous findings in largemouth bass [13]. Similar results have also been reported in yellow catfish (*Pelteobagrus fulvidraco*) [14] and rainbow trout [15], which confirm that the bioactive substances in TM could improve the antioxidant stress ability of fish. Moreover, this study also found that the expression of anti-inflammatory cytokines (*il-1β* and *tgf-β*) was significantly up-regulated and that of proinflammatory cytokines (*il-8* and *il-10*) was significantly down-regulated in the TM24 and TM36 groups with the increase in TM replacement level, suggesting that substitution of appropriate levels of FM with TM can regulate the inflammatory response of the liver. This may be due to the presence of bioactive substances such as chitin and tenecin 1 [40], an antimicrobial peptide, in TM at appropriate levels. Chitin has been reported to have the ability to recruit and activate innate immune cells and to induce the production of cytokines and chemokines through various cell surface receptors in yellow catfish [14] and gilthead sea bream [37]. In addition, the expression of protein metabolism genes (*pi3k*, *mtor*, *4ebp2* and *got*) was significantly up-regulated in the TM24 and TM36 groups as the level of TM substitution increased. The current findings suggest that an appropriate replacement level for TM in the diet can improve hepatic antioxidant capacity, effectively regulate the release of inflammatory mediators and alleviate the damage to cellular structure caused by inflammatory reactions, thereby improving liver health and the utilization efficiency of protein, and so promoting the growth of largemouth bass.

## 5. Conclusions

The present study found that dietary TM affected the growth performance and hepatic health of fish. The supplementation of dietary TM at appropriate levels (7.80%) could enhance antioxidant capacity and immunity, thus improving the growth performance of largemouth bass. However, the supplementation of dietary TM at high levels (21.23%) damages liver health and inhibits the growth of largemouth bass. The quadratic regression model analysis, based on WGR against dietary TM replacement level, indicated that the optimum replacement level was 19.52%. Thus, TM could be considered as a promising and suitable alternative protein source for largemouth bass.

## Figures and Tables

**Table 1:** Amino acid composition and nutrient levels of FM and TM.

Items	FM	TM
Essential amino acid		
Thr	2.70	2.59
Val	3.21	4.87
Met	1.82	1.04
Ile	2.72	3.29
Leu	4.74	5.61
Phe	2.50	3.62
His	2.16	0.52
Lys	4.90	3.71
Arg	3.55	4.14
Non-essential amino acid		
Asp	5.65	4.83
Ser	2.44	6.27
Glu	7.77	7.03
Gly	3.76	5.69
Ala	4.37	3.15
Tyr	2.20	3.05
EAA	28.3	29.39
Total amino acid	54.49	59.41
Nutritional levels		
Crude protein (%)	67.90	64.28
Crude fat (%)	10.55	3.44

**Table 2:** Composition and nutrient levels of experimental diets (DM basis).

Items	Groups				
	TM0	TM12	TM24	TM36	TM48
Ingredient (%)					
Peru steam fish meal	40.00	35.20	30.40	25.60	20.80
Domestic poultry by-product meal	10.00	10.00	10.00	10.00	10.00
*Tenebrio molitor* meal	0.00	5.28	10.56	15.85	21.13
Soybean meal	7.00	7.00	7.00	7.00	7.00
Soy protein concentrate	6.00	6.00	6.00	6.00	6.00
Cottonseed protein	5.00	5.00	5.00	5.00	5.00
Wheat gluten	3.00	3.00	3.00	3.00	3.00
Cassava starch	9.00	9.00	9.00	9.00	9.00
Fish oil	1.90	2.00	2.20	2.40	2.50
Soy lecithin	1.50	1.50	1.50	1.50	1.50
Soybean oil	4.00	4.00	4.00	4.00	4.00
Vitamin premix	1.00	1.00	1.00	1.00	1.00
Mineral premix	1.50	1.50	1.50	1.50	1.50
Choline chloride	0.50	0.50	0.50	0.50	0.50
Ca(H_2_PO_4_)_2_	1.50	1.50	1.50	1.50	1.50
Microcrystalline cellulose	8.10	7.45	6.69	6.03	5.28
Lys	0.00	0.04	0.08	0.12	0.16
Met	0.00	0.03	0.07	0.10	0.13
Total	100.00	100.00	100.00	100.00	100.00
Chemical composition (%)					
Crude protein	47.43	47.77	47.59	47.25	47.27
Crude lipid	13.68	13.71	14.10	14.15	13.76
Ash	10.44	10.83	10.40	10.11	9.67

Vitamin premix (mg kg^−1^ of diet): VA, 18; VD3, 5; VE, 150; VC (350 g kg^−1^), 500; VB1, 16; VB6, 20; VB12, 6; VK3, 18; riboflavin, 40; inositol, 320; calcium-D-pantothenate, 60; niacinamide, 80; folic acid, 5; biotin, 2; ethoxyquin, 100. Mineral premix (mg kg^−1^ of diet): Na, 30; K, 50; Mg, 100; Cu, 4; Fe, 25; Zn, 35; Mn, 12; I, 1.6; Se, 0.2; Co, 0.8.

**Table 3:** Primer pair sequences for real-time PCR.

Gene	Forward Primer (5′-3′)	Reverse Primer (5′-3′)	GenBank
*cat*	GGTGTTCACGGATGAGATG	GGAGAAGCGGACAGCAAT	XM_038704976.1
*sod*	GCGTGGGTAGATGGTTT	AGGGTTGATGGGCAGTA	XM_038713969.1
*gpx*	TGAGAAGGTGGATGTGAAT	GAAATGTCTGCTGTAGCG	XM_038697220.1
*pi3k*	TCTCAAGGGAGGAGGTCA	CCGAATGTCAGAGGGTC	XM_038723321.1
*mtor*	CAGCGACAGCGAGGTTG	GGGAAATGGAGCGGAAG	XM_038723321.1
*s6k1*	AGAATGTCTCTGACGACGAAC	ATCTGCTCTGCTCCTTTGT	XM_038708507.1
*4ebp2*	CATCTATGACCGTAAGTTCCTCCT	CATCTATGACCGTAAGTTCCTCCT	XM_038737859.1
*gpt*	GTGTATGCTGATGGTTGCC	TTGAGGTGGAATGGAAAGA	XM_038717755.1
*got*	GACCCTACCCAGGAGCAATG	GCGTCACGAGCCACAACC	XM_038737492.1
*il*-10	CAGCAGCATCATTACCACT	CAGAACCAGGACGGACA	XM_038696252.1
*tgf*	GGCAATGTAAGCGGTATGTC	CTTGGTGCTGTTGTAGAGGG	XM_038693206.1
*il*-1*β*	TGATGAGGGACTGGACC	ACTGTTGGCACGGATGT	XM_038733429.1
*il*-8	TTCTCCTGGCTGCTCTG	GGATGGCCCTCCTGTTA	XM_038704088.1
*tnf-α*	GACACCACCACTTCATCCA	AGCATCTTCTCCTCCATCA	XM_038723994.1

**Table 4:** ADC (%) in TM of dry matter, crude protein and crude lipid in largemouth bass.

Nutrients	ADC
Dry matter	74.66 ± 2.28
Crude protein	91.03 ± 1.86
Crude lipid	90.91 ± 1.87

**Table 5:** ADC (%) in TM of AA in largemouth bass.

Amino Acids	ADC of AA
Essential amino acid	
Thr	90.92 ± 0.10
Val	92.37 ± 0.28
Met	95.04 ± 0.02
Ile	93.27 ± 0.14
Leu	93.55 ± 0.16
Phe	94.69 ± 0.24
His	93.10 ± 0.62
Lys	96.00 ± 0.11
Arg	94.81 ± 0.24
Non-essential amino acid	
Asp	92.11 ± 0.01
Ser	94.68 ± 0.36
Glu	93.74 ± 0.10
Gly	91.30 ± 0.05
Ala	90.60 ± 0.29
Tyr	92.91 ± 0.50
EAA	93.86 ± 0.18
Total amino acid	92.89 ± 0.19

**Table 6:** Effects of fishmeal replacement with TM on the growth performance of *Micropterus salmoides* ^a^.

Items	Groups					*p*-Value		
	TM0	TM12	TM24	TM36	TM48	ANOVA	Linear Trend	Quadratic Trend
Initial body weight (g)	11.00 ± 0.00	10.93 ± 0.07	11.00 ± 0.00	11.00 ± 0.00	11.00 ± 0.00	0.452	0.496	0.563
Final body weight (g)	75.62 ± 0.53 ^b^	76.45 ± 0.66 ^b^	80.71 ± 1.19 ^c^	77.16 ± 0.45 ^b^	67.97 ± 1.83 ^a^	<0.001	<0.001	<0.001
WGR ^b^ (%)	587.48 ± 4.82 ^b^	599.35 ± 12.31 ^b^	633.75 ± 10.9 ^c^	601.47 ± 4.06 ^b^	517.90 ± 16.65 ^a^	<0.001	<0.001	<0.001
SGR ^c^ (%/d)	3.21 ± 0.01 ^b^	3.24 ± 0.03 ^b^	3.32 ± 0.02 ^c^	3.25 ± 0.01 ^b^	3.03 ± 0.05 ^a^	<0.001	<0.001	<0.001
PER ^d^	1.72 ± 0.01 ^a,b^	1.72 ± 0.03 ^a,b^	1.94 ± 0.01 ^b^	1.81 ± 0.05 ^a,b^	1.68 ± 0.09 ^a^	0.041	1.000	0.013
PRR ^e^ (%)	32.33 ± 0.30 ^a^	36.14 ± 0.27 ^b^	35.87 ± 0.50 ^b^	34.90 ± 0.21 ^b^	34.54 ± 0.80 ^b^	0.010	0.050	<0.001
LRR ^f^ (%)	74.87 ± 0.82 ^c^	67.95 ± 1.00 ^b^	65.93 ± 1.12 ^b^	59.02 ± 0.73 ^a^	67.27 ± 1.36 ^b^	<0.001	<0.001	<0.001
FR ^g^ (%/d)	2.50 ± 0.04	2.48 ± 0.02	2.37 ± 0.02	2.46 ± 0.02	2.47 ± 0.08	0.317	0.573	0.141
FCR ^h^	1.10 ± 0.01	1.08 ± 0.02	1.05 ± 0.02	1.10 ± 0.01	1.17 ± 0.07	0.268	0.206	0.075
SR ^i^ (%)	100.00 ± 0.00	100.00 ± 0.00	100.00 ± 0.00	100.00 ± 0.00	100.00 ± 0.00	1.000	1.000	1.000

^a^ Values with different letters represent significant differences in Tukey’s test (*p* < 0.05). ^b^ Weight gain rate (WGR) = 100 × [ final weight (g) − initial weight (g)]/initial weight (g). ^c^ Specific growth rate (SGR) = 100 × [ln (mean final weight) − ln (mean initial weight)]/77 days. ^d^ Protein efficiency ratio (PER) = total weight gain (g)/protein intake (g). ^e^ Protein retention ratio (PRR) = 100 × [total protein retention (g)/protein intake (g)]. ^f^ Lipid retention ratio (LRR) = 100 × [total lipid retention (g)/lipid intake (g)]. ^g^ Feeding ratio (FR) = 100% × total feed intake/[final weight (g) + initial weight (g)]/2 × t]. ^h^ Feed conversion ratio (FCR) = total feed intake (g)/weight gain (g). ^i^ Survival rate (SR) = 100 × (final number of largemouth bass/initial number of largemouth bass).

**Table 7:** Effects of fishmeal replacement with TM on body morphological measurements and body composition of *Micropterus salmoides* ^a^.

Items	Groups					*p*-Value		
	TM0	TM 12	TM 24	TM 36	TM48	ANOVA	Linear Trend	Quadratic Trend
**Morphological measurements**								
CF ^b^ (g/cm^3^)	2.33 ± 0.07	2.23 ± 0.05	2.39 ± 0.04	2.23 ± 0.05	2.29 ± 0.02	0.184	0.751	0.944
VSI ^c^ (%)	9.98 ± 0.62	8.67 ± 0.35	8.55 ± 0.33	8.53 ± 0.35	8.86 ± 0.79	0.282	0.164	0.097
HIS ^d^ (%)	2.63 ± 0.11 ^b^	2.31 ± 0.17 ^a,b^	2.11 ± 0.12 ^a,b^	2.13 ± 0.15 ^a,b^	1.85 ± 0.13 ^a^	0.009	0.001	0.583
**Body composition**								
Moisture (%)	68.35 ± 0.56	68.63 ± 0.35	69.41 ± 1.40	68.81 ± 0.78	69.34 ± 0.38	0.453	0.167	0.619
Crude protein (%)	17.55 ± 0.08 ^a^	19.66 ± 0.14 ^c^	18.88 ± 0.11 ^b^	18.91 ± 0.21 ^b^	18.67 ± 0.08 ^b^	<0.001	<0.001	<0.001
Crude fat (%)	10.57 ± 0.14 ^b^	10.02 ± 0.12 ^a,b^	9.67 ± 0.04 ^a^	9.46 ± 0.30 ^a^	9.33 ± 0.28 ^a^	0.009	0.001	0.225
Ash (%)	3.95 ± 0.06	3.66 ± 0.01	3.85 ± 0.09	3.98 ± 0.01	3.95 ± 0.10	0.208	0.535	0.085
Liver lipid content (%)	4.06 ± 0.06	4.69 ± 0.26	4.43 ± 0.50	4.43 ± 0.05	4.13 ± 0.59	0.727	0.936	0.206

^a^ Values with different letters represent significant differences in Tukey’s test (*p* < 0.05). ^b^ Condition factor (CF) = 100 × (body weight/body length^3^). ^c^ Viscerosomatic index (VSI) = 100 × viscera weight (g)/body weight (g). ^d^ Hepatosomatic index (HSI) = 100 × hepatic weight (g)/body weight (g).

**Table 8:** Effects of fishmeal replacement with TM on hepatic antioxidant capacity and protein metabolism in *Micropterus salmoides* ^a^.

Items	Groups					*p*-Value		
	TM0	TM12	TM24	TM36	TM48	ANOVA	Linear Trend	Quadratic Trend
Cat (U/mg prot)	7.26 ± 0.24 ^a^	8.59 ± 0.71 ^a,b^	11.59 ± 0.29 ^c^	10.19 ± 0.38 ^b,c^	7.17 ± 0.34 ^a^	<0.001	0.320	<0.001
T-Sod (U/mg prot)	255.71 ± 7.86	242.88 ± 1.08	257.71 ± 0.17	266.15 ± 14.72	254.06 ± 0.13	0.093	0.243	0.801
Gpx (U/mg prot)	43.74 ± 1.02 ^a^	53.21 ± 0.65 ^b^	54.12 ± 0.37 ^b^	53.69 ± 0.56 ^b^	41.68 ± 0.86 ^a^	<0.001	0.146	<0.001
MDA (nmol/mL)	0.79 ± 0.05 ^a^	0.75 ± 0.02 ^a^	0.70 ± 0.02 ^a^	0.76 ± 0.02 ^a^	0.96 ± 0.02 ^b^	0.001	0.004	<0.001

^a b c^ Values with different letters represent significant differences in Tukey’s test (*p* < 0.05).

## Data Availability

The data that support the findings of this study are available from the corresponding author upon reasonable request.
